# HLA-A29 Birdshot Retinochoroiditis in Its 5th Decade: Selected Glimpses into the Intellectual Meanderings and Progresses in the Knowledge of a Long-Time Misunderstood Disease

**DOI:** 10.3390/diagnostics11071291

**Published:** 2021-07-19

**Authors:** Marina Papadia, Carlos Pavésio, Christine Fardeau, Piergiorgio Neri, Philippe G. Kestelyn, Ioannis Papasavvas, Carl P. Herbort

**Affiliations:** 1 Istituto Auxologico Italiano IRCCS, 20095 Milano, Italy; marinapapadia@yahoo.com; 2 Moorfields Eye Hospital and Biomedical Research Centre, UCL, London EC1V 2PD, UK; cpavesio@aol.com; 3 Reference Center for Rare Diseases, Department of Ophthalmology, La Pitié-Salpêtrière Hospital, Paris-Sorbonne University, 75013 Paris, France; christine.fardeau@aphp.fr; 4 Uveitis Service, Cleveland Clinic Abu Dhabi, Abu Dhabi Island-59, United Arab Emirates; pg.neri@gmail.com; 5 Cleveland Clinic Lerner College of Medicine of Case Western Reserve University, Cleveland, OH 44195, USA; 6 Ghent University, 9000 Ghent, Belgium; philippe.kestelyn@ugent.be; 7 Retinal and Inflammatory Eye Diseases, Centre for Ophthalmic Specialised Care (COS), Montchoisi Teaching Centre, Rue Charles-Monnard 6, 1003 Lausanne, Switzerland; i.s.papasavvas@gmail.com

**Keywords:** HLA-A29 birdshot retinochoroiditis, fluorescein angiography, indocyanine green angiography, optical coherence tomography, clinicopathology, immunopathology, diagnostic criteria

## Abstract

The appraisal of HLA-A29 birdshot retinochoroiditis (BRC) was fraught with pitfalls and misunderstandings. Progress in investigational methods has led to better knowledge and management of the disease. Our aim was to assess some of the steps that have led to better characterisation of the clinical entity of BRC. We performed a literature search analysing the relevant progress in disease origin, investigational and imaging methods, clinicopathology and classification, diagnostic criteria and management. Following developments were judged essential in the better appraisal and understanding of the disease: (1) new immunopathological hypotheses regarding the role of endoplasmic reticulum peptidases, (2) the essential importance of HLA testing, (3) relevant imaging modalities among which indocyanine green angiography is crucial, (4) diagnostic criteria that allow early diagnosis and (5) need of an early prolonged, as well as aggressive treatment combining more than one immunosuppressive agent. Based on these findings it is now possible to better define BRC, an indolent however severe disease, unlike thought before, involving the choroidal stroma and the retina independently and concomitantly that can be diagnosed early thanks to indocyanine green angiography and should be treated early and relentlessly.

## 1. Introduction

HLA-A29 birdshot retinochoroiditis (BRC) was first described almost simultaneously in a January 1980 study by Ryan and Maumenee including 13 patients, [1] followed by a Kaplan and Aaberg one in December 1980, complementary to the Ryan and Maumenee article, reporting four cases [2]. However, Gass had presented earlier, in October 1979, an article describing a cohort of 11 patients, which was published in October 1981. [3]. The first report gave the name of birdshot retinochoroidopathy to the disease [1], while Gass called it vitiliginous chorioretinitis [3]. Classically, the disease is described in following terms: “*BRC can be defined as a chronic, bilateral, inflammatory condition, which affects the posterior segment of the eye with involvement of the choroid and retinal vessels*” [4]. “*The retinal involvement is characterised by a vasculitis involving both small capillaries and large retinal vessels. The choroidal involvement is a primary stromal choroiditis characterised by the typical oval cream-coloured depigmented areas in the posterior pole and mid-periphery*” [5]. 

The path to more accurate knowledge during the forty years since the disease has been identified has been tortuous, fraught with pitfalls and mystifications. We address here some of the intellectual wanderings, delays and misunderstandings that hampered the precise appraisal of BRC and highlight the significant and relevant developments having contributed to the better, comprehensive knowledge of this disease, which had a significant impact on how we manage and monitor it.

## 2. Materials and Methods

We conducted a selective review of the literature based on a PubMed search using the term “birdshot” Among the 451 articles the search retrieved, we then considered the articles dealing with “HLA-A29”, “investigations” and “imaging methods”, “clinicopathology”, “classification”, “diagnostic criteria” and “management”. The points analysed more precisely were: (1) evolution of the rating of the severity of the disease in early and subsequent years; (2) advances in immune-pathogenetic hypotheses; (3) the role of HLA-A29 in diagnosis; (4) imaging methods relevant in practice; (5) diagnostic criteria relevant in practice. 

## 3. Results

### 3.1. Misjudgement on the Severity of the Disease in Early Years after the Initial Articles Reporting the Disease, and Why the Condition Was Considered a Benign Disease

**Figure 1:**
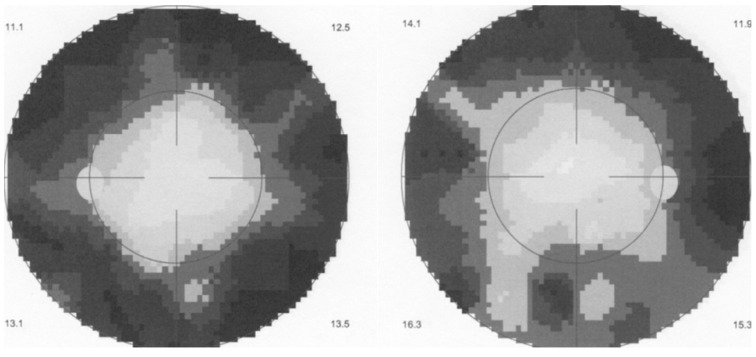
HLA-A29 birdshot retinochoroiditis. Bilateral quasi tubular visual field in a BRC patient still having a visual acuity of 1.0 ODS.

BRC was reported by all three first reports as an indolent slowly progressing disease that (1) had a “tendency to stabilise” or (2) that was found to maintain a relatively good visual acuity and all three reports as well as the extensive report by Priem and Oosterhuis in 1988 [6] voiced the impression of a probable unresponsiveness to corticosteroids. The apparent benign course of the disease and lack of need of systemic therapy was confirmed by further reports [7,8]. The problem was however that the main outcome criterion was visual acuity that is now known to remain relatively preserved for an extended period of time. On the other side, the appropriate parameter to follow the real progression of disease was found to be visual field testing, looking for scotomas to be performed routinely at each follow-up, and/or electrophysiology, when available, representing objective and precise biomarkers of the disease course [9,10,11,12,13]. Therefore, since progression of disease was based on visual acuity values, during the 2–3 decades after its first report, there was little incentive to recommend systematic immunosuppressive therapy for BRC, an attitude which was reflected in the uveitis textbooks of this period [14,15,16]. It became apparent that BRC produced severe impairment of visual function despite often retaining a relatively good visual acuity with patients presenting a tubular visual field and full visual acuity [9,17,18] (Figure 1) and no more than 15% of BRC patients seemed to have a low-grade benign course [19].

Thus, in a second phase, the deleterious evolution of BRC was recognised and several reports started to evoke the probable necessity for more systematic immunosuppression [20,21]. In 1988 and 1994, two among the first trials, showed that cyclosporin was beneficial in BRC [22,23]. In 2000, intravenous immunoglobulin (IVIg) treatment had a remarkable effect on BRC, measured with the more relevant criterion of visual field testing [24]. In 2005, a study reported the positive effect of non-steroidal immunosuppression on the outcome of BRC [25], and in 2009 the same institution achieved long-term control of inflammation by combining cyclosporin and mycophenolate mofetil [26]. The amount of evidence available steadily increased and, nowadays, indicates that aggressive and sustained immunosuppressive therapy is needed for the long-term treatment of BRC [27,28]. There is no specific agent that can be recommended but most conventional immunosuppressive [29,30,31] as well as biological agents [32,33,34] have been found to be efficient. The approach by trial and error has become possible as indocyanine green angiography was found sensitive for the monitoring of activity [35] giving rapid information on the efficacy or lack of efficacy of an agent in any given case, allowing fine-tuning of treatment [36]. Such an approach will allow to avoid the deleterious evolution that has been reported in ill- or non-treated patients [37].

### 3.2. Advances in Immunopathogenetic Hypotheses for BRC

Since its first description, BRC was extensively investigated, and significant progress has been made in the understanding of the immunological mechanisms of the disease. Although it is not the place in this clinical perspective to give a detailed account on the immunopathogenesis of the disease, it should be briefly cited. The topic has been thoroughly investigated by Kuiper’s group and others [38,39,40]. The presence of the HLA-A29 antigen is not the only predisposing factor to develop BRC. Indeed, HLA-A29 antigen is present in about 5–10% of the Western population and is identical in BRC cases and unaffected individuals [41] A genetic co-factor was identified explaining that only a fraction of HLA-A29 positive individuals develop BRC. On one hand, among HLA-A29 genetic polymorphism only a few HLA-A29 alleles are found in BRC patients, the principal allele being HLA-A29-02. On the other hand, there is the polymorphism in endoplasmic reticulum aminopeptidases (ERAP-1 and ERAP-2) trimming the peptide fragments before they are bound by class I HLA-A29 and presented to CD8+ T cells at the origin of autoimmune mechanisms in BRC. These combined polymorphisms confer a risk for disease which is substantially higher than the presence of one or the other polymorphism alone. We urge the reader to consult the detailed work by Kuiper and Venema, presenting the current stage of advancement of this issue in a recent publication which represents the most updated scientific and enlightening report on this crucial research topic in BRC [40].

### 3.3. The Essential Role of HLA-A29 in Diagnosis

According to the conclusion of an article entitled “HLA typing in uveitis: use and misuse”, HLA-testing was discarded as having limited usefulness as a diagnostic test in patients with uveitis [42]. Indeed, there are few uveitis conditions that benefit from the diagnostic help of HLA-testing, but they exist when they are performed in given settings. For instance, in Europe, the search for HLA-B27 is particularly useful to the clinician in case of a non-granulomatous anterior uveitis (NGAU). In such a geographical setting, over 63% of NGAU are HLA-B27 positive [43], which is extremely helpful both for the clinician and the patient. Indeed, in case of positivity, the clinician does not need to bother the patient with any additional test. It allows the clinician to consider the uveitis as a distinct clinical entity, the uveitis pattern and the treatment needed being clearly defined [44]. The other advantage for the clinician and the patient resides in the fact that the disease can be clearly explained to the patient as well as its potential evolution and steps to be taken. If HLA-B27 testing in NGAU is useful for diagnostic purposes, HLA-A29 testing in BRC is not only useful but has become a “sine qua non” criterion for diagnosis [45]. This association was first reported in 1982 [46]. Subsequent reports rated the association as high as 95% [41,47,48]. However, with the antibody testing methods false negatives are known to occur [49]. When PCR-based testing is used this association reaches close to 100%, if not 100%. Truly HLA-A29 negative BRC patients are a rarity if they exist at all when using the PCR method. No laboratory test in ophthalmology and even in medicine has such a high positivity rate and such a diagnostic value. Therefore, presence of HLA-A29 antigen should not only be an essential but a “sine qua non” criterion for the diagnosis of BRC. Nevertheless, in a very recent set of diagnostic criteria this crucial test was still not included as absolutely required [50], possibly because of adherence to the position that HLA typing has limited usefulness for diagnosis in uveitis [42].

In their inaugural report, Ryan and Maumenee stated “*the main reason for using birdshot retinochoroidopathy as a term for this entity is that this descriptive clinical picture does not imply a specific pathogenesis. Thus, when the pathogenesis has been elucidated, a more exact name can be applied rather than changing a pre-existent name with an implied pathogenesis.*” The time to complete the eponym has now come, although the pathogenesis is still not exactly known. The constant presence of HLA-A29 leads to the need to include HLA-A29 itself in the name of the disease. In general, once a disease name becomes adopted it is extremely difficult to change, which does not need to be done here. A few years ago, our group and others suggested to better define the disease by including HLA-A29 in its name, calling it HLA-A29 uveitis or better HLA-A29 birdshot retinochoroiditis to emphasise the extreme importance played by the HLA-A29 antigen [45]. 

### 3.4. Imaging Methods Relevant in Practice for Diagnosis and Follow-Up of BRC

The imaging investigation of inflammation of the posterior segment has made substantial advances in the last three decades. A distinction must however be made between modalities which are immediately relevant for the diagnosis and the follow-up of a given disease and those which are interesting for investigational research but ill-adapted and/or not sufficiently standardised to be relevant for diagnostic and/or follow-up purposes. Some of the new imaging modalities may be technically very sophisticated but are inappropriate for investigation of structures they are not designed for.

**Figure 2:**
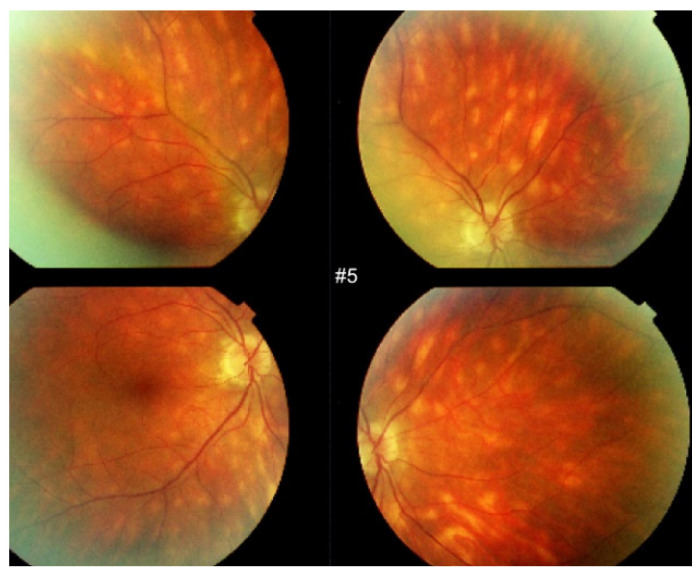
HLA-A29 birdshot retinochoroiditis. Rice shaped cream coloured birdshot lesions.

Fundus photography (FP) is important to document the rice-shaped cream-coloured birdshot lesions (RSCCBL). However, as the diagnosis is increasingly achieved before RSCCBL appear [51], the fundus may be normal and remain normal if early and sustained treatment is applied and FP might become useful to document lesions that break through despite treatment (Figure 2). 

#### 3.4.1. Fluorescein Angiography

**Figure 3:**
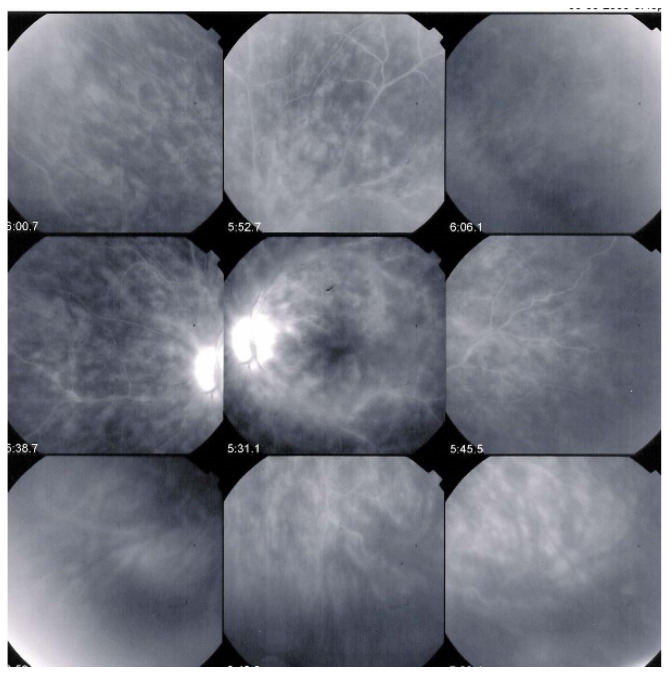
HLA-A29 birdshot retinochoroiditis. Diffuse retinal vasculitis involving vessels of all sizes, retinal oedema with relative sparing of the central macula and optic disc hyperfluorescence.

BRC involves independently both the retina and the choroid and fluorescein angiography (FA) is one of the two methods of choice, with retinal optical coherence tomography (OCT) to analyse and follow retinal disease [52,53]. It is the retinal involvement that causes the major part of disease morbidity, characterized by a profuse leakage due to retinal vasculitis involving all calibres of vessels (arterioles, capillaries and veins) (Figure 3).

**Figure 4:**
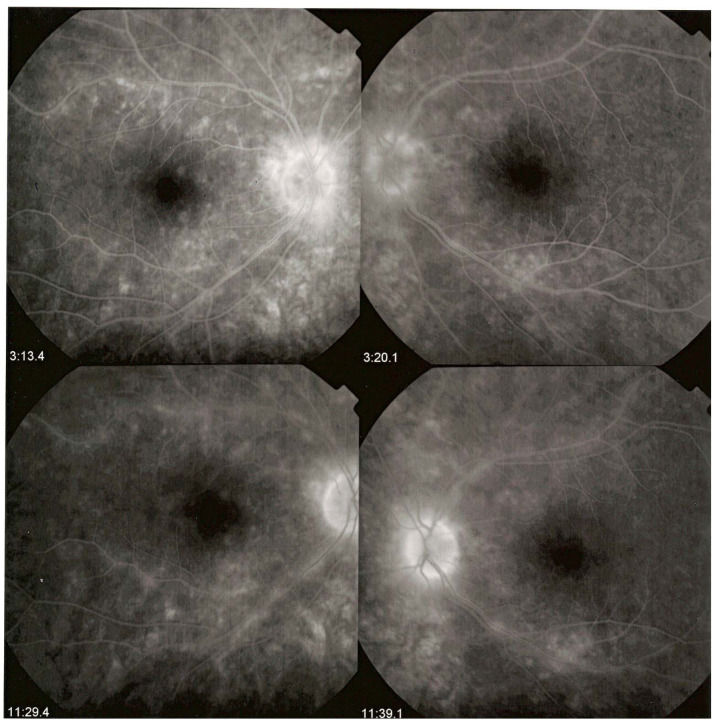
FA in HLA-A29 birdshot retinochoroiditis. Disc hyperfluorescence, retinal vasculitis, diffuse posterior pole oedema with relative sparing of the central macula.

The consequence of such a diffuse vasculitis is retinal and macular oedema. However, the foveola may be spared for a prolonged period of time which explains why visual acuity can remain relatively preserved even in advanced disease [54] (Figure 4).

**Figure 5:**
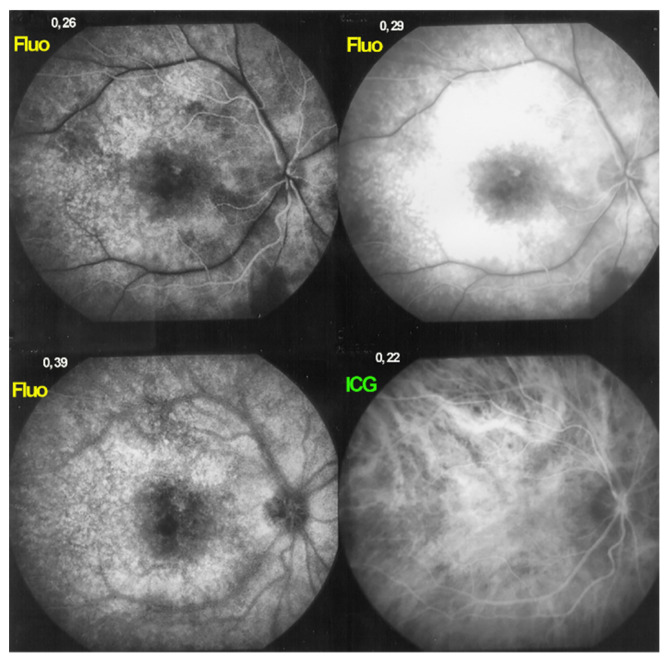
HLA-A29 birdshot retinochoroiditis, massive retinal exudation. There is such global retinal leakage (top right) that there is never fluorescein marking of the large veins (fluo pictures). This does however not correspond to arteriovenous perfusion delay, as the ICG frame already shows venous filling after 22′′, meaning no delay.

In some cases, the exudation from retinal vessels is such, that the large veins are never completely marked by the fluorescein. Gass noticed this feature and attributed it to arterio-venous circulation delay [3] (Figure 5).

**Figure 6:**
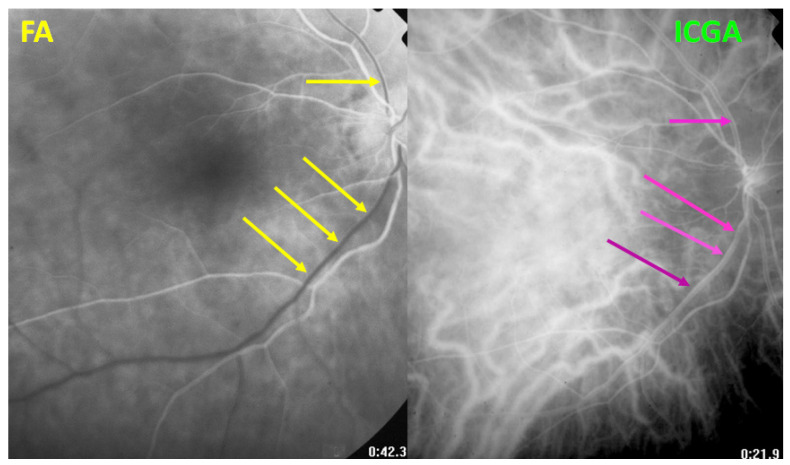
HLA-A29 birdshot retinochoroiditis. Close-up on discrepancy between FA and ICGA imaging of large veins. After 42′′, on FA, the veins are still not marked by fluorescein (yellow arrows); however, large veins are marked on the ICGA frame (crimson arrows) after 21.9 s.

This is not the case, since, when the arterio-venous times are observed on ICGA, large veins are marked in a normal timeframe, as the large ICG molecular complex cannot egress from the retinal vessels [55]. Optic disc hyperfluorescence is a constant finding on FA (Figure 6).

The initial exudative stage of retinal disease, if left untreated, evolves to an atrophic and thinned stage with filiform retinal vessels. For the follow-up of retinal involvement in BRC, FA is essential as it gives a global overview on the evolution of retinal vasculitis and can be quantified by an angiographic scoring system [56].

#### 3.4.2. Optical Coherence Tomography

##### Retinal SD-OCT

**Figure 7:**
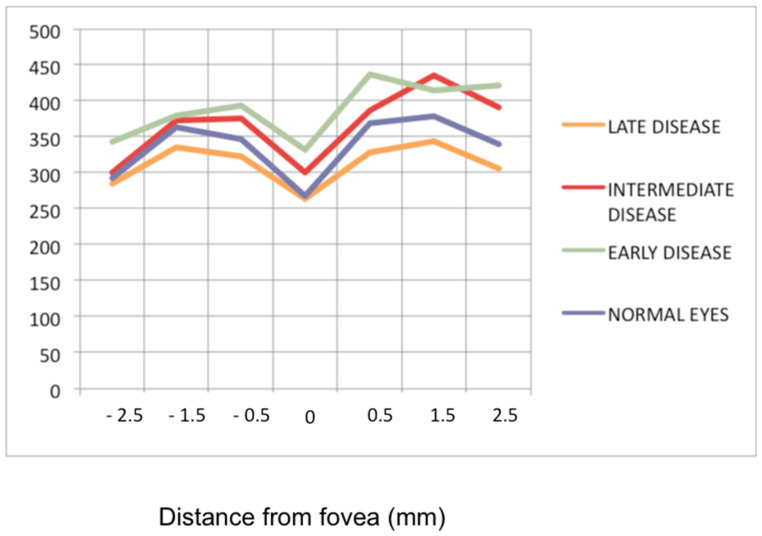
Progression of retinal disease as followed by optical coherence tomography (*x* axis indicates distance from fovea (=0); *y* axis indicates thickness of retina in microns). Early disease (green line) features thickening of the retina (and to a lesser degree the fovea). In late disease (yellow line) there is diffuse thinning except in the fovea; foveal thickness remains comparable to normal (purple line) (from Ophthalmic Surg Lasers Imaging 2012; 43-suppl S25–31).

Although spectral-domain OCT (SD-OCT) gives only information on the posterior pole, it allows another approach to the evolution of the retina in BRC and can be repeated easily, as it is not an invasive method. It gives information on the retinal thickness and on whether macular oedema is present as well as on its evolution. It was shown that in early exudative retinal disease the macular retina was thickened, but less so in the fovea [53]. Furthermore, foveolar thickness has been shown to remain more stable during the evolution of retinal disease [53] in line with the fact that the central macula is less hyperfluorescent than the rest of the posterior pole on FA. SD-OCT demonstrate the evolution from the exudative stage to the atrophic stage in more precise fashion, going from a thickened retina to a mixed stage of areas of thickened retina and thinned atrophic areas (Figure 7). Fine epiretinal membranes with moderate tractions were found in 93% of analysed cases [53]. Another application of OCT reported was the possibility to monitor retinal vasculitis, the major drawback being however that it lacks the global image obtained by FA [57].

##### Choroidal Enhanced-Depth Imaging OCT (EDI-OCT)

**Figure 8:**
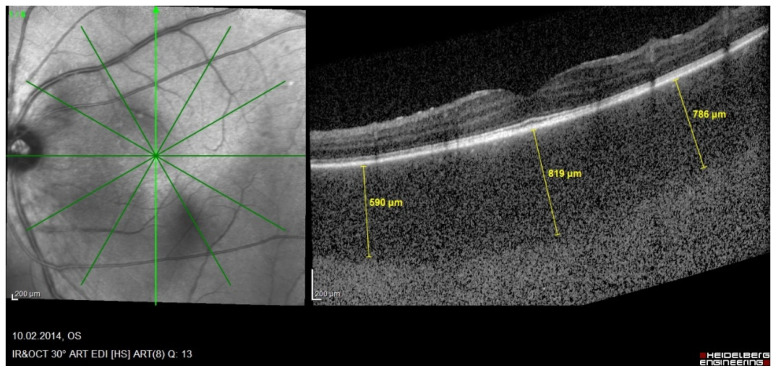
HLA-A29 birdshot retinochoroiditis; EDI-OCT. Substantial thickening of the choroid in an initial-onset BRC.

**Figure 9:**
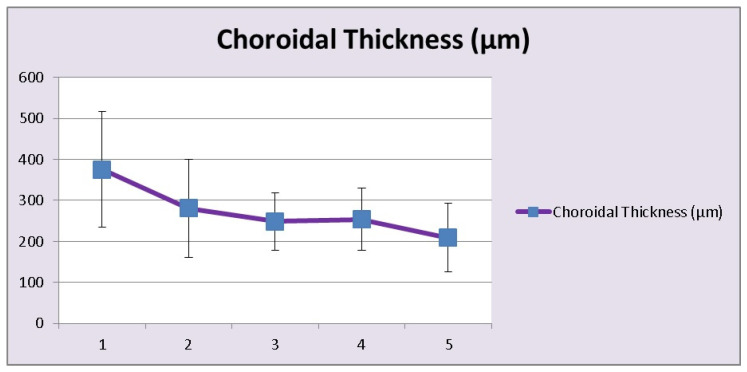
HLA-A29 birdshot retinochoroiditis; choroidal thickness. Over time, as disease progresses choroidal thickness is decreasing (1 = <1 year; 2 = 1–4 years, 3 = 5–9 years, 4 = 10–15 years, 5 = >15 years).

EDI-OCT is a non-invasive imaging modality making it possible to analyse the choroidal involvement in BRC. Depending on the stage of the disease choroidal thickness is either increased (in early disease) or decreased (in late, ill-treated disease). In early disease EDI-OCT clearly shows increased choroidal thickness which was shown to progressively decrease with time and in the absence of appropriate treatment, due to atrophy following uncontrolled disease for a long time [58] (Figure 8 and Figure 9).

Choroidal volume and thickness changes detected by EDI-OCT were shown to give useful information on choroidal inflammation in BRC [59]. During follow-up of disease to detect choroidal recurrence, EDI-OCT is more difficult to interpret as reactivation occurring in a previously thinned area may be missed. The information provided is of lesser quality than that obtained by indocyanine green angiography (ICGA) [60,61]. Despite its limitations, not showing the globality of the choroid, and not always detecting signs of activity, EDI-OCT still represents a valuable imaging method for BRC, having the advantage of being non-invasive.

#### 3.4.3. Indocyanine Green Angiography (ICGA) Is Essential for Choroidal Involvement and Early Diagnosis

**Figure 10:**
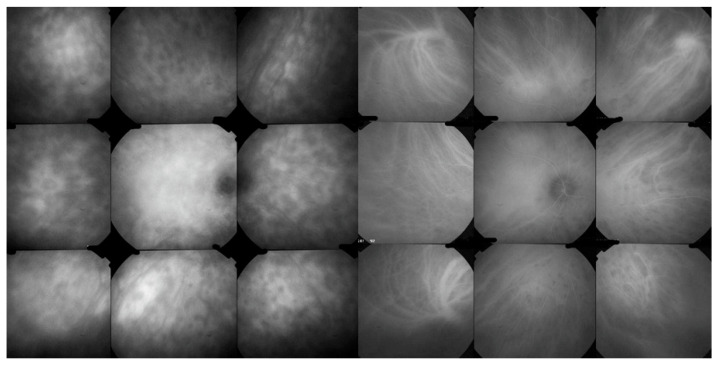
ICGA signs in BRC. Numerous HDDs in the pre-treatment panorama (**left**) that have disappeared after introducing immunosuppressive therapy (**right** panorama).

ICGA is the most appropriate imaging modality to assess choroidal inflammatory lesions in stromal choroiditis entities which include BRC [62]. The advantage of ICGA is the fact that it can detect and follow occult choroidal lesions globally throughout the fundus, not accessible otherwise [63]. Indeed, EDI-OCT shows diffuse thickening of the choroid in initial-onset BRC but limited to the posterior choroid and unable to show individual lesions in the same way as ICGA does [64]. We standardized ICGA findings in BRC back in 1999 [65]. ICGA characteristic features in stromal choroiditis that includes BRC are mainly (1) regular round hypofluorescent dark dots (HDDs) evenly scattered in the whole fundus indicating choroidal stromal foci and (2) fuzziness of vessels indicating choroidal vasculitis [66] (Figure 10).

**Figure 11:**
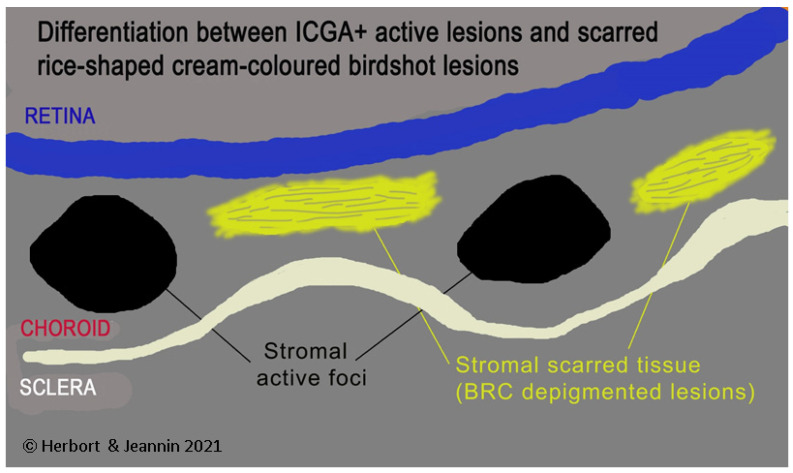
HDDs versus birdshot lesions. Cartoon inspired by Joan Mirò explaining that HDDs seen on ICGA do not correspond to rice shaped cream coloured birdshot lesions which do not appear on ICGA.

**Figure 12:**
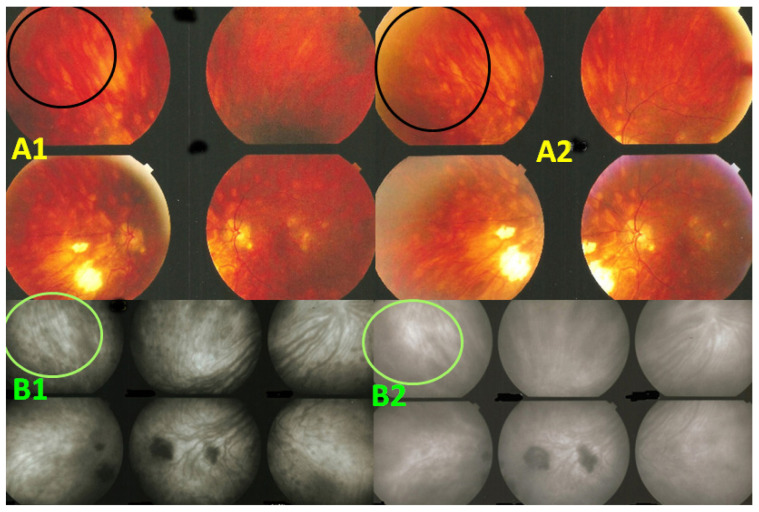
Indocyanine green angiography (ICGA) lesions do not correspond to birdshot retinochoroiditis (BRC) fundus lesions. This man was 68 years old at presentation; his history featured a 32-month diagnostic delay and a 38-month treatment delay (left eye). Fundus images before treatment (**A1**, left quartet of fundus images) reveal well-delineated BRC fundus lesions, as well as whitish chorioretinal scars, inferior-nasally. After 3 years of inflammation suppressive treatment (IST), the fundus aspect exhibited absolutely no change (**A2**, right quartet). ICGA frames reveal numerous typical hypofluorescent dark dots (HDDs), as well as a larger hypofluorescent chorioretinal atrophic scar temporal to the fovea, before treatment (**B1**, left 6 frames). Three years after IST, HDDs had disappeared and chorioretinal atrophy exhibited the same geographic hypofluorescent area (**B2**, right 6 frames). This example demonstrates that HDDs do not correspond to BRC fundus lesions, which are angiographically silent and do not respond to treatment as they are choroidal scars.

HDDs are caused by stromal inflammatory foci as was confirmed by a histopathological report [67]. Many BRC HDDs become isofluorescent on late angiographic frames indicating that granulomas do not occupy the full thickness of the choroidal stroma and don’t touch the choriocapillaris which was also shown by histopathology. [67] It has been reported that HDDs are the angiographic expression of BRC fundus lesions [68] which is incorrect (Figure 11). HDDs resolve after the introduction of therapy which is not the case of rice shaped cream coloured birdshot lesions (RSCCBLs), likely because BRC fundus lesions correspond to areas of depigmentation that no longer have any active inflammation once the stromal pigment islets have been “digested”, resulting from the immune reaction against the stromal melanocytes. This explains why BRC fundus lesions are angiographically silent on ICGA but they allow to see the fluorescent sclera through the choroidal depigmented patches on late FA frames [69]. On the contrary, the round nodular lesions located in the choroidal stroma are not visible on fundus examination because they are below the RPE (Retinal pigment epithelium) (Figure 12).

Presence of diffuse retinal vasculitis on FA associated with HDDs on ICGA should lead the clinician to search for the presence of the HLA-A29 antigen which confirms the diagnosis of BRC when present. The crucial importance and determining role of ICGA for BRC resides in the fact that it allows to diagnose the disease before birdshot lesions appear [51]. Moreover, if thanks to ICGA, diagnosis is achieved early before RSCCB lesions are seen and appropriate immunosuppressive therapy is initiated early and applied in a sustained way these lesions will never develop [51]. Despite its undisputed and obvious advantages, ICGA is still reluctantly used in some places and publications that mention ICGA are scarce. To date, of the 451 publications listed in the PubMed database on BCR, only 36 articles mention ICGA, of which one third comes from our group. 

#### 3.4.4. Additional Imaging Methods Not Directly Relevant for Diagnosis and Practical Follow-Up

More recently, newer imaging methods have been applied to BRC, which have some research interest but are not directly relevant for diagnostic and follow-up purposes either because they are not standardised or because they are not appropriate and therefore cannot give information on structures they are technically not supposed to analyse. Two modalities that have been applied to many clinical situations including BRC are fundus autofluorescence (FAF) and OCT-angiography (OCT-A). Blue-light FAF (BAF) is a very useful modality for diseases involving the RPE or the choriocapillaris. BCR does not involve the RPE or choriocapillaris until a later stage of the disease and therefore FAF is ill-suited for this condition. Indeed, there are only a few studies that studied FAF in BRC. They showed areas of hypoautofluorescence indicating that the disease can involve secondarily the RPE/choriocapillaris complex [70,71]. The signification of FAF findings is difficult to interpret as lesions depend on the stage and evolution of the disease and are not essential for routine practical purposes but can give information on the stage of the disease. There are even less studies devoted to OCT-A in BRC. This method might be applied to analyse the status of retinal superficial and deep capillary plexuses, an analysis far from being standardised. It could be used to detect choriocapillaris/RPE drop-out and for inflammatory choroidal neovascularisation.

### 3.5. Practical Diagnostic Criteria for Early Diagnosis

In 2006 a group of clinicians published a consensus set of diagnostic criteria for BRC [72]. However, these criteria raised concerns about accuracy, completeness, and data interpretation. In 2017, a group of European BRC specialists, having seen more than 360 BRC patients, made a critical review of them by pointing out the flaws and incongruencies of such a consensus report [45]. Very recently, the authors of the 2006 consensus report, under a different label, issued another set of diagnostic criteria, called classification criteria, the complexity of which results from a sophisticated epidemiological, “machine learning” and statistical enterprise [50]. Improvement over the previous work was the inclusion this time of ICGA and the fact that HLA-A29 was given a more rightful importance. While this work may represent a thorough intellectual effort, it fails to mention, in the discussion, the existence of other approaches on the topic, and is not practical for the use by the clinician. What is needed from diagnostic criteria is the pioneering pragmatism that was clearly expressed by the European BRC specialists [45]. The clinician must have simple and practical criteria that allow for a rapid and secure diagnosis in order to initiate prompt and appropriate management of the disease. This was the goal sought by the European criteria, abandoning the multifocal choroiditis criterion, which is still the centrepiece of the new attempt in the recent publication [50]. The European-based approach focuses on giving importance to: (1) imaging techniques that allow to detect early BRC signs before multifocal choroiditis is seen, and (2) to the unavoidable search for HLA-A29 antigen. These European-based criteria (Table 1) represent a user friendly, powerful tool for timely diagnosis, while the new proposed criteria still do not allow to make a diagnosis before multifocal choroiditis is present, a moment when the disease has already been evolving for months if not years, missing the necessary therapeutic window of opportunity in order to avoid irreversible histological damages. 

## 4. Management

Since the editorial published in the *British Journal of Ophthalmology* in 2017 [45] not much has to be added about the management of BRC. Inflammation suppressive treatment, including steroidal and non-steroidal immunosuppression, must be introduced early even before the development of multifocal choroiditis, as soon as functional signs are detected by visual field testing, the most sensitive functional test. This is possible with the European-based criteria. It was even shown that “birdshot lesions” never appear when treatment is started early [51]. If involvement is asymmetrical, local periocular treatment by sub-Tenon’s depot corticosteroid injections can be tried. However, this obviates the use of systemic immunosuppression in only 15% of patients [19]. Immunosuppression mostly necessitates more than one agent and must be prolonged. Conventional immunosuppression is classically used, without clear evidence of superiority for one agent over another. The management is often based on trial and error, being guided by ICGA monitoring to assess efficacy or not of a given combination of immunosuppressants [35,36]. Indeed, one approach may function for one patient and not for another. Biologic agents represent additional therapeutic possibilities and have been shown to be useful [32,33,34]. However, for these therapies also, only trial and error will guide the clinician. Sustained therapy was able to prevent thinning of the choroid [58], improve visual field deficits [29], retinal vasculitis [25,52] and HDDs [52]. The duration and tapering of therapy also are points that remain unanswered. We know when to start, but it is hard to know when to stop. The therapy should probably be prolonged for years. We noted recurrences each time we attempted to stop treatment, which was also reported by others [73].

## 5. Conclusions

Despite hesitations, delays and misunderstandings in the knowledge of this still enigmatic disease, we are able today to understand its course and behaviour. We can diagnose it early, treat it efficiently and monitor it precisely, which is encouraging at the dawn of the 5th decade since its discovery.

## Figures and Tables

**Table 1:** European based simplified diagnostic criteria for BRC * [45].

1. Presence of vitritis in one or both eyes (required)
2. Presence of retinal vasculitis in one or both eyes (required)
3. Stromal choroiditis, as evidenced by ICGA, in both eyes (required)
4. HLA-A29 antigen positivity (required)
5. Visual field anomalies in one or both eyes (supportive)
6. Absence of extra-ocular inflammatory site (supportive)
7. Presence of rice-shaped depigmented “birdshot lesions” (BRC fundus lesions) (strongly supportive but not required)

* Allowing also early diagnosis before multifocal choroiditis is seen.
